# Subjective versus objective symptom intensities ratings in cervical dystonia and hemifacial spasm across a botulinum neurotoxin cycle

**DOI:** 10.1002/brb3.2023

**Published:** 2021-01-05

**Authors:** Julia Wöllner, David Weise, Bernd Leplow

**Affiliations:** ^1^ Department of Psychology Martin‐Luther‐University Halle‐Wittenberg Halle (Saale) Germany; ^2^ Department of Neurology Martha‐Maria Hospital Halle‐Dölau Halle (Saale) Germany; ^3^ Department of Neurology University of Leipzig Leipzig Germany; ^4^ Department of Neurology Asklepios Fachklinikum Stadtroda Stadtroda Germany; ^5^ Institute of Therapy and Health Research IFT‐Nord Kiel Germany

**Keywords:** botulinum neurotoxin, dystonia, hemifacial spasm, symptom severity rating

## Abstract

**Background:**

Subjective symptom complaints often do not match the expert’s ratings in focal dystonia. Nonetheless, perceived symptom intensities drive compliance and outcome of botulinum neurotoxin (BoNT) treatment.

**Methods:**

Perception of symptom development across a BoNT cycle was obtained in 21 cervical dystonia (CD) and 15 hemifacial spasm (HFS) patients at four time points during a BoNT cycle. Subjective assessments were recorded by means of a quality‐of‐life questionnaire and a patient diary containing items related to subjective severity of disease, mood, pain, social impairment, and quality of life. Medical investigation used the Tsui score and TWSTRS, and a HFS rating score, respectively.

**Results:**

In both patient groups, subjective intensities were strongly associated with psychological variables. Only in CD did objective assessment moderately correlate with subjective ratings solely at the beginning and the end of the BoNT cycle. Overall, the beneficial effects of BoNT treatment were only loosely associated with subjective experiences in both groups.

**Conclusion:**

The emotional situation should be assessed regularly in patients undergoing BoNT therapy.

## Graphical abstract

Our study revealed that there was only moderate and no correlation of subjective and objective rating of symptom severity in cervical dystonia and hemifacial spasm, respectively, during a therapy cycle of Botulinum neurotoxin. Of note, no correlation was found at the intermediate time points when Botulinum neurotoxin therapy is supposed to work. However, subjective intensities were strongly associated with psychosocial variables in cervical dystonia and hemifacial spasm.

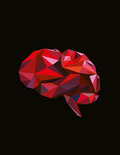

## 1 INTRODUCTION

Primary cervical dystonia (CD) is the most common form of focal dystonia. The resulting motor symptoms are the most pronounced attributes of CD and influence the degree of disability. (Ben‐Shlomo et al., 2002) Due to the resulting pain and movement restrictions, CD is a highly stigmatizing and disabling condition associated with an enormous functional impairment in a patient’s daily life. (Dool et al., 2016; Hall et al., 2006; Papathansiou et al., 2001; Rinnerhaler et al., 2006; Tomic et al., 2016) CD‐related bad postures, tense facial expressions, and compensatory postures lead to a socially conspicuous appearance. (Fabbrini et al., 2010; Guendel et al., 2001; Kim et al., 2016; Ozel‐Kizil et al., 2008; Rinnerhaler et al., 2006) As a result, social situations are avoided frequently with harmful consequences for the satisfaction with BoNT treatment. (Leplow et al., 2017; Skogseid et al., 2007)

There is rising evidence that nonmotor symptoms, such as pain, depression, or anxiety, play a pivotal role in quality of life (QoL) and treatment success. (Ben‐Shlomo et al., 2002; Fabbrini et al., 2010; Jankovic & Orman, 1984; Mueller et al., 2002; Werle et al., 2014) The fact that psychiatric disorders arise before the onset of the movement disorder underlines the possibility of a shared pathophysiology. (Contarino et al., 2016; Fabbrini et al., 2010; Smit et al., 2016) The clinical picture of CD depends on not only neuropathology, but also psychological factors, which means that objective therapeutic improvements do not automatically result in corresponding improvements in a patient’s QoL. (Ben‐Shlomo et al., 2002; Dool et al., 2016; Leplow et al., 2017; Yang et al., 2017)

Treatment of CD with locally injected botulinum neurotoxin (BoNT) is the first choice for the reduction in symptoms. BoNT injections are usually administered approximately at a three‐month interval (BoNT cycle), with the maximum effect after four weeks and decreasing benefit within the next 6–8 weeks. The therapy effect is usually assessed by a patient’s self‐report and possibly using clinical scales at the end of a BoNT cycle (when BoNT is not supposed to have an effect anymore). Hence, the adaption of BoNT therapy is mainly based on patients’ reports. However, the adequate assessment and comparison of subjective and objective effectiveness during a BoNT cycle are usually not feasible in clinical practice.

Thus, we compared the evolution of subjective and objective symptoms and their perception across a BoNT cycle. Thereby, we addressed the question of whether objective rating of symptom severity matches subjective experience in patients with CD compared to hemifacial spasm (HFS), a disorder of the peripheral nervous system with muscle contractions of facial muscles, which is also treated with BoNT, but characterized by a lower frequency of psychiatric comorbidities. (Fabbrini et al., 2010; Smit et al., 2016; Yang et al., 2017)

## 2 PATIENTS AND METHODS

### 2.1 Study population

Twenty‐one CD (19 female, age: 57.1 ± 14.9 years) and 15 HFS (13 female, 69.9 ± 13.5 years) patients were included in the study during the consultation of the outpatient clinic at the Department of Neurology of the University Hospital Leipzig from February to August 2018. Fifteen CD patients were treated with the preparation abobotulinumtoxin, six with incobotulinumtoxin, 14 HFS patients with abobotulinumtoxin, and one HFS patient with onabotulinumtoxin. The study was approved by the Ethics Committee of the University Hospital Leipzig (Reference No.: 014/18‐ek). The study was in accordance with the Declaration of Helsinki, and written informed consent was obtained from each patient.

### 2.2 Study design

Both patient groups were examined at four time points: At t1, the BoNT injection took place (start of the BoNT cycle), t2 was assessed four weeks later (anticipated maximum effect), t3 happened two weeks before the regularly planned upcoming injection, and t4 was scheduled while BoNT reinjection was reperformed (end of the BoNT cycle). At t1, objective assessment of severity was supplemented by a semi‐structured clinical interview. Furthermore, a QoL questionnaire (see below) and a self‐rating scale for depression and anxiety screening (hospital anxiety and depression scale in the German version, HADS‐D) were applied, respectively.

### 2.3 Survey methods

Objective assessment of severity of CD was carried out using the Tsui scale and the Toronto Western Spasmodic Torticollis Rating Scale (TWSTRS). In the case of HFS, a severity score was used based on the Jankovic Rating Scale. (Jankovic & Orman, 1984) The HFS score can take values from 0 (*no symptom severity*) to 4 (*strong symptom severity*). The Craniocervical Dystonia Questionnaire (CDQ‐24) was used to investigate the health‐related QoL. Due to the lack of an adequate disease‐specific procedure for recording the health‐related QoL of HFS patients, the CDQ‐24—adapted in formulation—was performed in this group. Higher values are associated with a poorer QoL.

**FIGURE 1:**
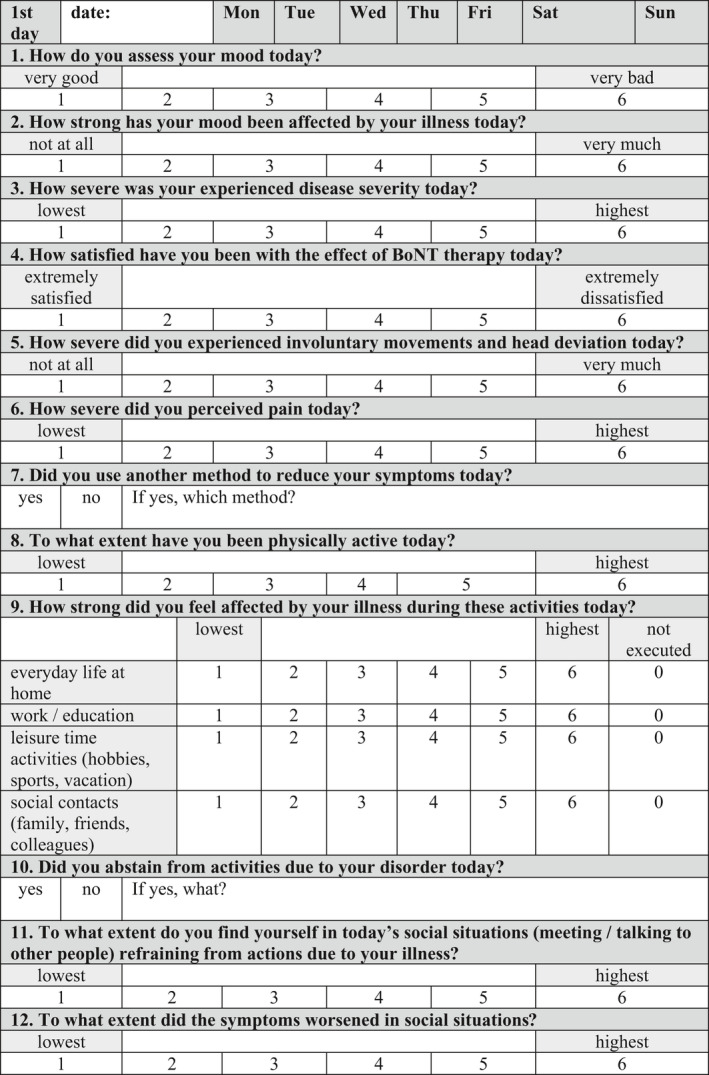
Patient diary daily rating (translation)

Patients of both groups kept a self‐developed diary for the daily recording of subjective severity of disease, mood, pain, social impairment, and restrictions in everyday activities across the entire BoNT cycle (t1–t4; Figure 1). Proven items from Leplow et al (Leplow et al., 2017) were used, which were answered on a six‐level ranking scale based on the German school grade scaling system (1 = *best*, 6 = *worst*). Using this scale, perceived symptom severity was assessed by the mean score of item 3 (“*How severe was your experienced disease severity today*?”) and 5 (“*How severe did you experience involuntary movements or head deviation today*?”). Pain was separately asked about by item 6 (“*How strong did you perceive pain today*?”). Moreover, satisfaction with BoNT therapy was investigated by item 4 (“*How satisfied have you been with the effect of BoNT therapy today*?”).

### 2.4 Statistical analysis

For statistical analysis of the two (groups)‐by‐four (time points of assessment) design, a sample size calculation was performed that revealed 16 patients in each group. As we wanted to detect only large effects, we chose an effect size of 0.40, a power of 0.95, and an alpha level of 0.05 (two‐tailed). Scores were taken from the diary at the respective time points (t1–t4). Rank correlations (Spearman’s rho) were calculated to assess the relationship between objective and subjective severity assessments. Values were indicated as mean value ± SD. Depending on the requirements for statistical procedures, either nonparametric (Mann–Whitney *U* tests, *t* tests) or parametric tests were used for group comparisons and comparisons across times of assessment (ANOVA, Friedman’s tests), respectively (see results).

## 3 RESULTS

Clinical baseline data did not differ between groups. In CD patients, disease duration was 10.4 ± 9.2, in HFS patients 7.8 ± 4.9 years (*U* = 119.00, Z = −.26, *p* = .79). Injection intervals (CD 11.4 ± 1.7, HFS 11.4 ± 1.8 weeks; *U* = 148.00, *Z* = −.31, *p* = .76) and duration of BoNT therapy (CD 8.0 ± 8.2, HFS 7.9 ± 6.2 years; *U* = 119.00, *Z* = −.26, *p* = .79) were similar in both groups. No significant difference was found regarding satisfaction with BoNT treatment (*U* = 97.00, *Z* = −1.87, *p* = .10), HADS depression (*U* = 115.00, *Z* = −1.18, *p* = .25), or HADS anxiety (*U* = 133.00, *Z* = −.57, *p* = .59).

**FIGURE 2:**
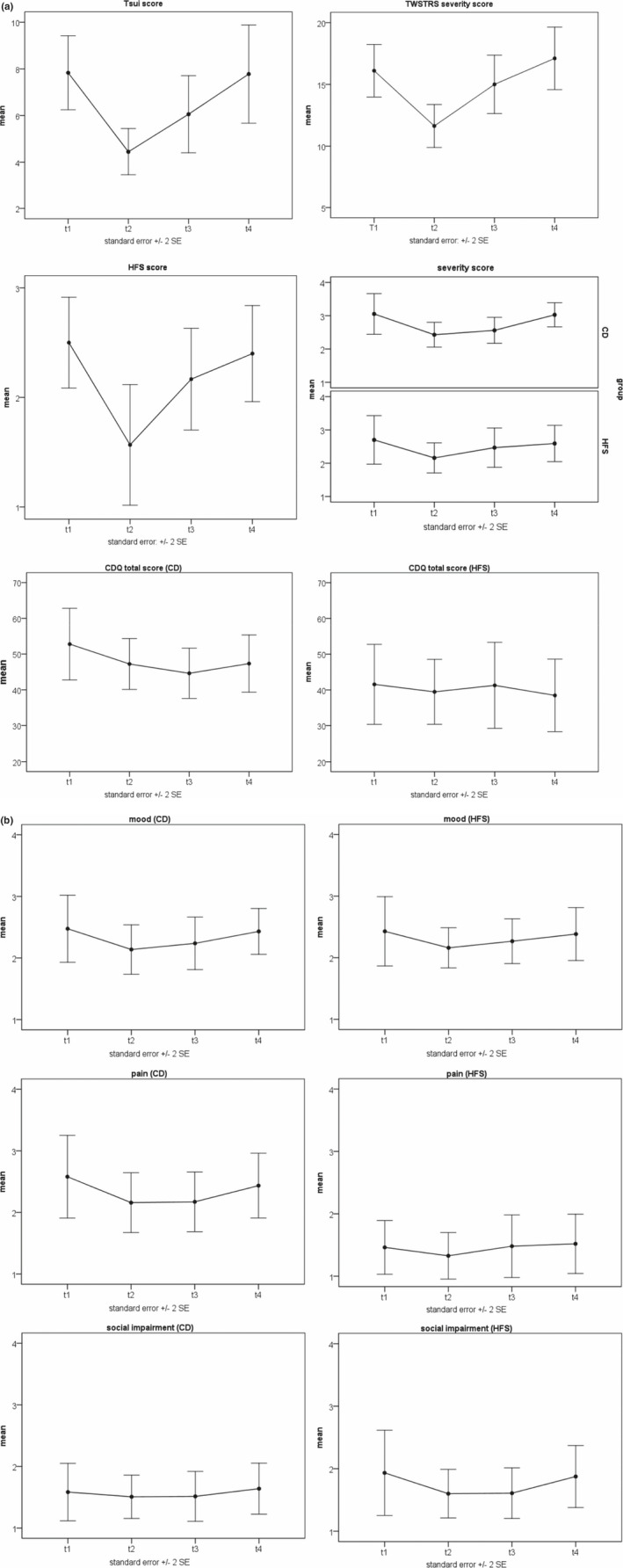
(a) Tsui scale, Toronto Western Spasmodic Torticollis Rating Scale (TWSTRS), hemifacial spasm (HFS) severity score, subjective symptom severity score (based on the patients’ diary), and Craniocervical Dystonia Questionnaire (CDQ) at every time point in patients with cervical dystonia (CD) and HFS. (b) Scales for mood, pain, and social impairment (based on the patients’ diary) at every time point in both patient groups

Figure 2a shows that objective severity ratings followed the well‐known U function in patients undergoing BoNT treatment. On the contrary, with subjective severity ratings in both CD and HFS patients, the BoNT cycle is less pronounced. ANOVA with repeated measurements revealed that objective symptom severity, as measured by the Tsui score, yielded significant differences across the four points of measurement (*F*(3,51) = 14.47, *p* < .01, eta = 0.46). Equivalent results were obtained by the TWSTRS severity score (*F*(3,54) = 28.10, *p* < .01, eta = 0.61), the HFS severity score (*F*(1.6, 22,43) = 8.45, *p* < .01, eta = .38), the CDQ total score in CD patients (*F*(3,51) = 4.28, *p* = .01, eta = 0.20), and subjective symptom severity ratings (*F*(2,11, 37,98) = 5.787, *p* = .01, eta = 0.24) in CD patients, respectively. In HFS patients, neither subjective symptom severity nor CDQ total score revealed significant differences between points of measurement.

Contrary to these results, there was not a significant difference between points of measurement for mood, pain, social impairment, or any one of the CDQ subscales. Planned comparisons between measurements showed significant differences between most of the points of measurement, especially in CD patients (Table 1).

**TABLE 1:** Planned post hoc comparisons between points of measurement

Variable	t1/t2	t1/t3	t2/t3	t2/t4	t3/t4
TWSTRS severity score	*p* < .001	*p* = .483	*p* < .001	*p* < .001	*p* = .003
Tsui score	*p* < .001	*p* = .012	*p* = .048	*p* = .003	*p* = .089
HFS score*	*p* = .004	*p* = .453	*p* = .270	*p* = .049	*p* = .174
Severity patient diary (CD)*	*p* = .123	*p* = .116	*p* = 1.00	*p* = .013	*p* = .013
Severity patient diary (HFS)	*p* = .474	*p* = 1.00	*p* = .741	*p* = .381	*p* = 1.00
CDQ‐24 total score (CD)	*p* = .393	*p* = .086	*p* = 1.00	*p* = 1.00	*p* = .763
CDQ‐24 total score (HFS)	*p* = 1.00	*p* = 1.00	*p* = 1.00	*p* = 1.00	*p* = .814

All coefficients are Bonferroni‐corrected.

* Greenhouse–Geisser correction (degrees of freedom)

Only at t1 and t4 was the subjective severity assessment of CD patients moderately correlated with objective assessments (Table 2). However, in HFS patients, there was no correlation between objective and subjective assessments at all (Table 2). By contrast, subjective intensities were strongly associated with psychosocial variables, such as mood or social impairment, for both CD and HFS patients (Table 2). All correlations showed that high subjective symptom severity was associated with reduced quality of life, enhanced experience of stigma, negative mood, reduced daily functioning, and increased social impairment, respectively. This pattern of result was seen in both patient groups.

**TABLE 2:** Severity ratings and subjective psychosocial variables (rank correlations (Spearman’s rho))

Variable	Subjective severity rating (patient diary)			
	t1	t2	t3	t4
CD patients				
TWSTRS severity score	0.43	−0.05	0.37	0.47*
Tsui score	0.50*	−0.10	0.37	0.43
Mood (patient diary)	0.48*	0.80^**^	0.63^**^	0.70^**^
Pain (patient diary)	0.84^**^	0.73^**^	0.71^**^	0.65^**^
Social impairment (patient diary)	0.29	0.67^**^	0.79^**^	0.45*
CDQ‐24 (total score)	0.74^**^	0.75^**^	0.72^**^	0.50*
CDQ‐24 (activities of daily living scale)	0.89^**^	0.72^**^	0.80^**^	0.61^**^
CDQ‐24 (stigmatization scale)	0.50*	0.63^**^	0.54*	0.34
HFS patients				
HFS score	0.03	−0.21	0.29	0.27
Mood (patient diary)	0.92^**^	0.83^**^	0.85^**^	0.76^**^
Pain (patient diary)	0.82^**^	0.38	0.57*	0.74^**^
Social impairment (patient diary)	0.85^**^	0.83^**^	0.44	0.48
CDQ‐24 (total score)	0.52*	0.77^**^	0.52	0.46
CDQ‐24 (activities of daily living scale)	0.45	0.48	0.70*	0.27
CDQ‐24 (stigmatization scale)	0.51	0.56*	0.46	0.55*

Abbreviations: CD, cervical dystonia; HFS, hemifacial spasm; TWSTRS, Toronto Western Spasmodic Torticollis Rating Scale; CDQ‐24, craniocervical dystonia questionnaire (adapted for HFS patients).

* *p* < .05; ^**^ *p* < .01.

## 4 DISCUSSION

We could show that the subjective perception of disease severity is not mainly driven by objective motor symptoms, but is primarily determined by a number of psychological variables, such as QoL in both CD and HFS (Table 2). Another core result is that the undoubtedly beneficial effects of BoNT treatment are not reflected by subjective experiences in the two patient groups.

BoNT treatment yielded the expected positive effect on motor function—as long as objective assessments are undertaken (Figure 2a). (Weiss et al., 2017) On the contrary, none of the psychological variables, such as mood, pain experience, or social impairment, yielded any differences (Figure 2b). Instead of displaying the expected U‐shaped curve, no differences at all were seen between the four points of measurement. Those lacks of differences may indicate “perceptional traits,” which have been developed over years while living with a disabling disorder. If this interpretation is valid, ratings about mood, pain perception, and the social situation should be relatively independent from the motor situation.

This may be the reason why correlation coefficients between objective and subjective assessments were only moderately high in CD if symptom severities were high, as was the case either at the beginning or at the end of the BoNT cycle. Moreover, no correlation was found at the intermediate time points when BoNT therapy is supposed to work. In HFS, even no correlation between objective and subjective assessments was found at any time point. On the contrary, correlations between subjective symptom intensities and psychological variables are consistently high in both patient groups. This is what has to be expected if perception of motor function dissociates from the patient’s psychological status.

Such a dissociation is highly relevant for the evaluation of therapeutic effects not only in clinical studies, but also in clinical routine, when treatment adjustments (e.g., regarding dosage or injected muscles) are made based on the patient’s assessment, especially between two BoNT injections, when the patient is not routinely seen. In other words, as long as we do not combine subjective ratings and objective assessment, we do not really know whether BoNT has worked on motor symptoms during two injections.

It is well accepted that in CD (more than in HFS patients), psychiatric comorbidities—mainly depression and anxiety—are very frequent. (Dool et al., 2016) Subjective disease severity perception seems to be mainly driven by psychological factors rather than measures of objective motor symptoms. This might be the reason for missing correlations of motor and nonmotor symptoms in previous studies. (Hall et al., 2006) Hence, these comorbidities have to be taken more thoroughly into consideration in the clinical management of CD and HFS patients. Objective severity of CD and HFS patients at t1 (Table 2) was in line with several other clinical studies. (Contarino et al., 2016; Kim et al., 2016) Moreover, the lack of correlation between objective symptom assessment and perceived symptom intensities perfectly fits with recent studies. (Contarino et al., 2016; Smit et al., 2016; Yang et al., 2017) All these studies showed that QoL was not primarily associated with (motor) symptom severity, but with other factors, such as pain, disability, and psychiatric burden, especially depression. (Fabbrini et al., 2010; Skogseid et al., 2007; Weiss et al., 2017) Furthermore, it was shown that premorbid emotional disturbances and missing coping strategies predict poor satisfaction with treatment outcome. (Leplow et al., 2017) Stigma, embarrassment in social situations, and psychophysiological activation, which, in turn, leads to symptom exacerbation followed by avoidance behavior, lead to a vicious circle that determines not only QoL, but also compliance to BoNT therapy. This might be the reason why extensive complementary and alternative medicine methods are as frequent in CD, especially in patients with low satisfaction with BoNT and low QoL. (Junker et al., 2004; Viehmann et al., 2014)

As satisfaction with BoNT therapy, compliance to medical treatment, coping behavior, and QoL depend on psychological factors more than the objective status of motor functioning, these factors need closer attention. Interestingly, this consideration seems to be valid for both CD and HFS patients. This is especially remarkable because in CD, nonmotor symptoms, especially depression, are believed to be primarily part of a shared pathophysiology. (Fabbrini et al., 2010) From BoNT studies in poststroke spasticity, also a disorder of the central nervous system, such a high impact of psychiatric comorbidities on the therapeutic effect and QoL is not known and has not been investigated systematically.

One would therefore hypothesize that subjective changes in symptom severity that are driven by mental disorders, such as depression, would correlate with objective assessment in CD rather than in HFS. However, psychiatric burden and numerous psychological variables seem to be potent general confounders or drivers of motor disease severity, which are widely independent of the specific disease. We therefore recommend that these factors be carefully considered, for example, by means of a brief structured interview addressing emotional burden, distress, impairment of quality of life, experience of stigma and its related states of symptom exacerbation, quality of life, and alterations of disease perception (Figure 1). These questions should be combined with simple, well‐anchored rating scales (see methods section) with respect to both the evaluation of clinical trials and patients’ management of daily practice, at least when BoNT therapy is supposed to be adapted. Furthermore, drug treatment or psychotherapeutic interventions should be considered in patients with mental stress or illness.

## CONFLICT OF INTEREST

The authors declare no potential conflict of interests.

## AUTHOR CONTRIBUTION

JW acquired the data, analyzed and interpreted the data, drafted the manuscript, and approved the final version of the manuscript to be published. DW conceived and designed the manuscript, acquired the data, analyzed and interpreted the data, drafted the manuscript, critically revised the manuscript for important intellectual content, and approved the final version of the manuscript to be published. BL conceived and designed the manuscript, analyzed and interpreted the data, drafted the manuscript, critically revised the manuscript for important intellectual content, and approved the final version of the manuscript to be published.

### Peer Review

The peer review history for this article is available at https://publons.com/publon/10.1002/brb3.2023.

## Data Availability

The data that support the findings of this study are available from the corresponding author upon reasonable request.
